# L-lysine and surfactant-assisted synthesis of NiCo bimetal oxides for electrochemical water splitting

**DOI:** 10.1016/j.isci.2024.110823

**Published:** 2024-08-31

**Authors:** Anila Tabassum, Sadia Ata, Norah Alwadai, Wissem Mnif, Abid Ali, Abid Ali, Arif Nazir, Munawar Iqbal

**Affiliations:** 1 School of Chemistry, University of the Punjab, Lahore 54590, Pakistan; 2 Department of Physics, College of Sciences, Princess Nourah bint Abdulrahman University, P.O. Box 84428, Riyadh 11671, Saudi Arabia; 3 Department of Chemistry, Faculty of Sciences at Bisha, University of Bisha, P.O. BOX 199, Bisha 61922, Saudi Arabia; 4 Department of Allied Health Sciences, The University of Chenab, Gujarat 50700, Pakistan; 5 Department of Chemistry, The University of Lahore, Lahore 54590, Pakistan

**Keywords:** Natural sciences, Chemistry, Applied sciences

## Abstract

In the present study, bimetallic oxides comprising nickel (Ni) and cobalt (Co) were synthesized using a facile hydrothermal method in the presence of CTAB and L-lysine. Their efficacy in catalyzing hydrogen production under alkaline conditions was assessed. Structural, vibrational, and morphological characteristics were analyzed utilizing X-ray diffraction (XRD), Fourier transform infrared (FTIR) spectroscopy, and scanning electron microscopy (SEM) techniques. The SEM images revealed a needle-like shape which is due to the surfactant addition. The NiCo oxides exhibited the lowest onset potential of 83 mV for HER and 130 mV for OER under standard conditions. The catalysts needed a potential of 286 and 450 mV to attain a current density of 50 mA/cm^2^ along with Tafel slope values of 119 and 332 mV/dec for HER and OER, respectively. These results suggested that L-lysine as a surfactant is highly effective in the fabrication of NiCo bimetal oxides for electrolytic water splitting applications.

## Highlights

• L-lysine-assisted Ni/Co bimetallic oxide has been synthesized by hydrothermal method
• Surface morphology and crystal structure are examined through SEM and XRD analysis
• Ni/Co catalyst is employed for hydrogen production under an alkaline medium
• L-lysine-NiCo is promising for electrolytic water-splitting applications

## Graphical abstract

**Figure:**
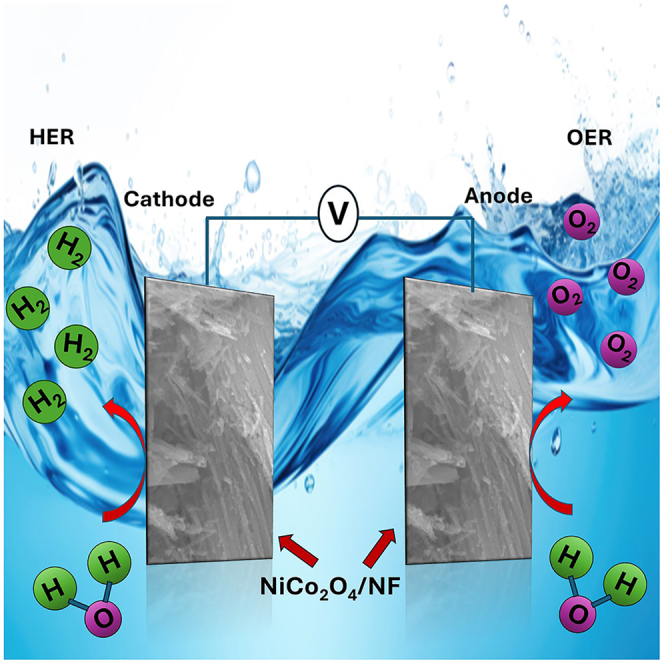

## Introduction

Electrolytic splitting of water into hydrogen and oxygen is considered a vital tool to reserve sporadic renewable energy entailed to sustain world advancement, hydrogen being plentiful and having escalating calorific value appraised as a green fuel with only a clean way of production through electrolysis.^1^^,^^2^^,^^3^ Overall water electrolytic reaction comprising hydrogen evolution (HER: ${2H}^{+}+{2e}^{-}\to H_{2}$) and oxygen evolution (OER: ${2H}_{2}O\;\to \;{4H}^{+}+{4e}^{-}+O_{2}$) transpired at the cathode and anode, respectively, requires a high potential of 1.23V to progress appreciably.^4^^,^^5^ Until now, noble metals Pt and Ir/Ru-based materials, recognized as benchmark catalysts for HER and OER which on account of being costly, instability, and scarcity hampers their practical implementation.^6^ Moreover, the HER performance is greatly influenced by the pH of the medium while at the industrial level water electrolysis is typically employed in alkaline solutions.^7^ For that reason, strenuous efforts have been made to explore modest and highly efficient non-noble metals-based electro-catalysts working under a basic medium.

Furthermore, during gas evolution, gas bubble formation obstructs electrolyte diffusion, increases resistance, and impedes mass transfer leading to substandard performance. Therefore, materials constructed at the nanoscale are highly interesting because of their small particle size along with huge surface area which enhances conductivity by efficient charge and mass transfer.^8^^,^^9^ The nanoscale of the particle size spans from 1 to 100 nm. The term nanotechnology was first coined by a Nobel laureate named Richard Feynman in the annual meeting of the American Physical Society (1959). Until the 1980s, discussions about nanotechnology were limited, but afterward, it gained significant momentum, offering enormous advancements in materials fabrication and their applications. Thus, this uniqueness of nanomaterials makes them efficient in every perspective.^10^ Extended further, nanocatalysts are usually pasted on a conductive substrate utilizing a polymeric binder (Nafion) which buries active sites and lessens the contact between electrolyte and catalyst. Under large applied potential, this coating may shed down leading to the leaching of the catalyst thus lowering the performance on the whole. So, this problem can be addressed by employing self-supported catalysts directly grown on the substrate and ensuring rapid charge transmission.^11^

Currently, first-row transition metals based on sulfides, chalcogenides, carbides, phosphides, and oxides (hydroxides) are studied extensively to substitute traditional noble metal-based materials.^12^ Typically, a higher potential of water electrolysis deteriorates the structure and composition of these materials, so transition metal oxides are ascertained to be compatible with the reaction because of much resistance to corrosion and alkalinity of the medium. Furthermore, transition metal oxides are also considered promising candidates for water splitting owing to their switching of oxidation states and intermixing to form composites with additional synergistic effects.^13^^,^^14^^,^^15^ Transition metal oxides of Fe, Co, and Ni are explored to a great extent by their low cost, higher activities, and abundance. Also, studies revealed that multi (bi or tri) metallic oxides such as Co-Fe, Ni-Fe, Ni-Co, and Co-Ni-Fe are more dynamic for splitting of water than their monometallic systems.^16^^,^^17^ The synergy of bimetallic materials modulates the electronic structure and active sites of the catalysts. During the hybridization of metal d-states, significant changes occur in the electronic states due to electron transfer.^18^

In general, Ni and Co-based electro-catalysts having Ni^+3^ and Co^+3^ oxidation states are contemplated as highly efficient for showing appropriate adsorption energies for H_2_O under moderate conditions.^19^ In this regard, bi-metallic NiCo_2_O_4_ was found to be a potential candidate as a bifunctional material for HER and OER simultaneously under an alkaline environment and has been employed as an electro-catalyst since the 1970s. NiCo_2_O_4_ has a spinel inverse structure with a face-centered cubic lattice where Ni occupied octahedral sites along with Co at both tetrahedral and octahedral sites ensuring better performance than its mono metallic NiO/Co_3_O_4_ counterparts. The mixed redox couple of Co^+3^/Co^+2^ and Ni^+3^/Ni^+2^ with multi-oxidation states aids in enhancing electronic conductivity and capacity to interact with water molecules. In addition, these metal ions in their multiple oxidation states are highly stable in NiCo_2_O_4_ thus further confirming its capability for redox reaction eventuated in water electrolysis.^20^^,^^21^^,^^22^^,^^23^ Motivated by these features, various methodologies such as electrodeposition, sol-gel, and hydrothermal have pertained to fabricating NiCo_2_O_4_ nanomaterials including nanoparticles, nanotubes, nanosheets, and nanocomposites but still they encounter reduced intrinsic electronic conduction.^24^

Therefore, it is challenging to fabricate nanomaterials with the desired structure, shape, and size. The surfactants contributed a major role in this regard as surface-directing agents by controlling morphology, particle size, porosity and structural variations. The surfactants also halted the agglomeration of the nanosized materials.^25^ Due to their hydrophilic/hydrophobic confirmation, the surfactant molecules arranged themselves into a variety of assemblies, thus resulting in various shapes and imparting spectacular properties in enhancing the performance of the nanomaterials. The cetyltrimethylammonium bromide (CTAB) is a cationic surfactant and has been utilized largely in the fabrication of nanoparticles as reported in the literature.^26^ On the other hand, amino acids manifested various benign environmental characteristics based on their non-irritated green nature, antibacterial properties, exceptional solubility, and biodegradability. Adding more, the zwitterionic nature of the amino acids (presence of both amino and carboxyl group) is beneficial in surface functionalization of the nanomaterials. For that reason, amino acids have been exploited widely for the fabrication of nanosized particles and their surface modifications.^27^

Based on the aforementioned facts, the present study was conducted for the synthesis of Ni-Co (NiCo_2_O_4_) bimetal oxides with the aid of amino acid and surfactant, which has not been reported previously, and their water splitting efficiency was compared. The fabrication of Ni-Co oxides was performed using CTAB and L-lysine through a one-pot hydrothermal method. The prepared catalysts were characterized by XRD, FTIR, SEM, and EDX techniques.

## Results and discussion

### Properties of the materials

**Figure 1:**
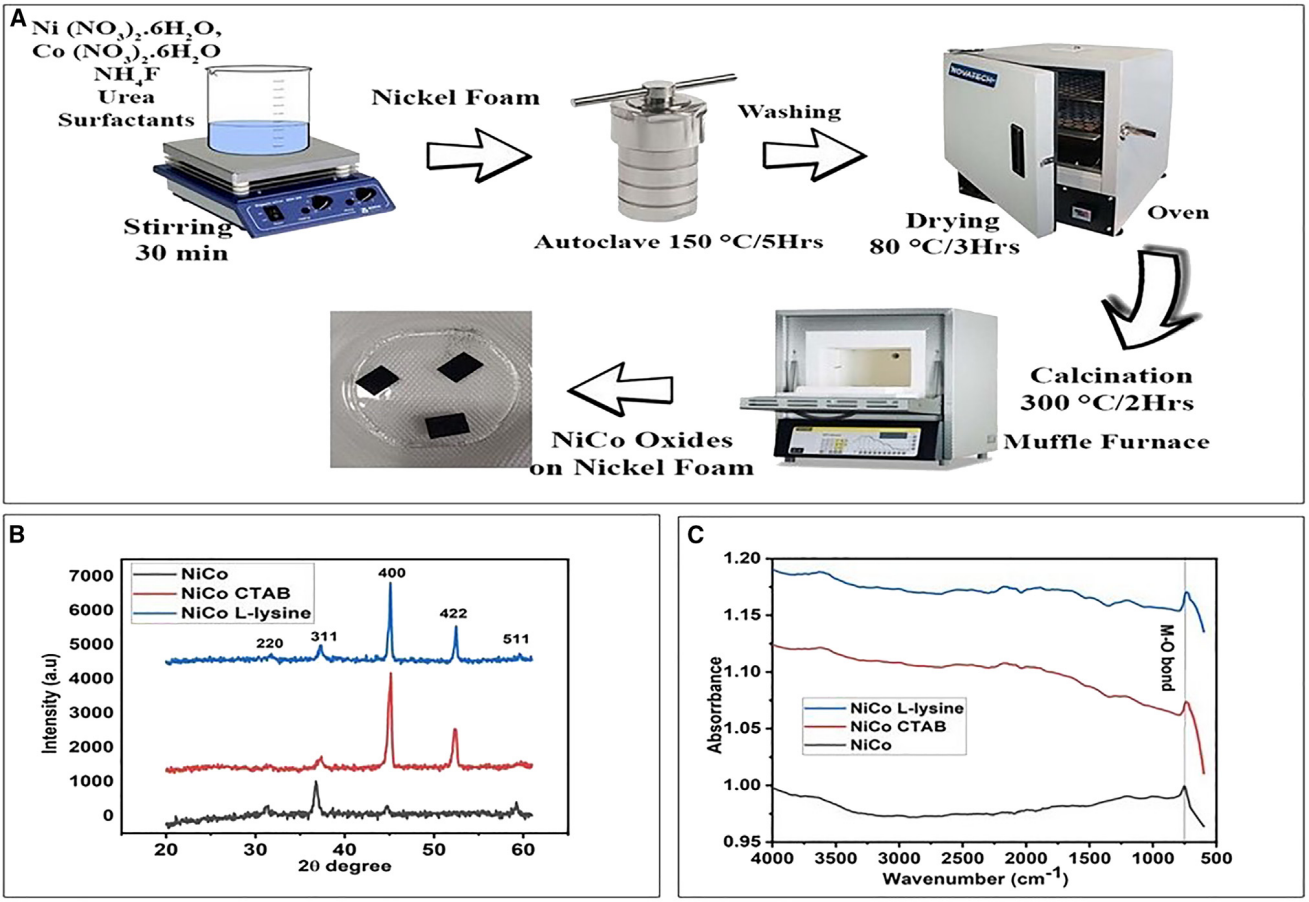
The synthesis scheme, XRD and FTIR analysis of the prepared NiCo oxides (A) Schematic for the synthesis of NiCo oxides by hydrothermal method. (B) XRD pattern of NiCo oxides. (C) FTIR spectrum of NiCo oxides.

The crystalline structure and purity of phase for NiCo, NiCo CTAB and NiCo L-lysine have been assessed by XRD pattern (Figure 1B) Nickel foam peaks have also been observed to play a role by enhancing intensity.

The XRD data for all the samples is well correlated with JCPDS card no. 20–0781 delineated cubic spinel NiCo_2_O_4_ phase structure devoid of any impurity. The intense peaks located at 31°, 37°, 45°, 52°, and 59° correspond to Miller indices planes (220), (311), (400), (422) and (511) respectively. Furthermore, the presence of different surfactants (CTAB, and L-lysine) imparts very little peak fluctuations along with peak shifting which is mostly attributed to inner strain and surface structure. The Scherrer formula (Equation 1) was applied to calculate crystallite size for the prepared nanocatalysts.

(Equation 1) $$D=\frac{K\lambda }{\beta coss\theta }$$

Here, D denoted average crystallite size in nm, K is a constant as 0.94, λ is an X-ray wavelength emitted from Cu source (Cu Kα = 0.1546 nm), β is full width at half maximum (FWHM) in radians, and θ is the peak position or diffraction angle. All the intense peaks were considered for measuring the size. The average crystal size was computed as 15, 11, and 10 nm for NiCo, NiCo CTAB, and NiCo L-lysine, respectively. These results manifested size reduction with the addition of the surfactants. Here L-lysine assisted NiCo oxides displayed smaller size in comparison to other nanocatalysts thus ensuring their higher activity.^28^^,^^29^

The microstrain (ε) of NiCo oxides was measured using the following expression (Equation 2).

(Equation 2) $$\varepsilon =\frac{\beta }{4}\;\tan\;\theta$$

In the dominion of nanomaterials, the term microstrain also referred to as lattice strain mainly emerges by shifting of atoms from their local positions owing to structural imperfections like grain boundaries, stacking faults and dislocations. These deviations cause the broadening of diffraction peaks leading to compression and expansion of surface atoms without destroying their local position. In real, the strain, chemical composition and synergism have a direct influence on the activity of any catalyst by altering the adsorption energies of intermediates. Based on the XRD pattern results, the microstrain was found to be 0.055, 0.151 and 0.119 for NiCo, NiCo CTAB and NiCo L-lysine, respectively (Table 1). The microstrain depends on the crystallite size of the nanoparticles. A reduction in crystallite size implies an elevation in microstrain values, which in turn suggests an improvement in electro-catalytic activity. These results are well correlated with the literature on the subject.^30^

**Table 1:** The structural parameters of NiCo oxides

Samples	Lattice Constants (a = b = c) (Å)		Cell Volume (Å)	Size (nm)	Dislocation Density (nm^−2^)	Micro-strain (%)
	Theoretical	Calculated				
NiCo	8.11	8.097	530.85	15	0.0044	0.055
NiCo CTAB	8.11	8.027	517.20	11	0.0083	0.151
NiCo L-lysine	8.11	8.036	518.94	10	0.01	0.119

From crystallite size, dislocation density (δ) can also be determined. The dislocation density can be defined as the number of dislocation lines (defects/irregularities) per unit area of the crystal. These crystal defects disrupt the properties of the materials. The expression for dislocation density is given by the formula shown in Equation 3.

(Equation 3) $$\delta =\frac{1}{D^{2}}$$

There is an inverse relationship between dislocation density and crystallite size. As results demonstrated, the dislocation density increased with a decrease in crystallite size from 0.0044 to 0.01 for NiCo-to-NiCo L-lysine (Table 1). Consequently, this increment of dislocation density facilitates the creation of defects in the bimetallic oxides, thereby assisting in improving their catalytic performance.^31^^,^^32^

The cubic structural parameters as unit cell length (lattice constants a, b and c) along with volume were computed using Equations 4 and 5 ^33^ and data is depicted in Table 1.

Equation 4) $$a=d\;{\left(h^{2}+k^{2}+l^{2}\right)}^{1/2}$$

(Equation 5) $$V=a^{3}$$

Here, *h*, *k*, and *l* represented the Miller indices of the reflected planes while a, d, and V denoted unit cell lengths, interplanar distance and volume, respectively. All the calculations were carried out utilizing a single (hkl) plane (400). The observed values of the lattice parameters for NiCo, NiCo CTAB and NiCo L-lysine are in good agreement with the literature.^34^

The FTIR spectrum for NiCo, NiCo CTAB and NiCo L-lysine were shown in Figure 1C. Occasionally, spinel oxides exhibited two strong peaks in the range of 400–800 cm^−1^ commensurate to metal-oxygen bonds in tetrahedral and octahedral regions. Here in this pattern ranges from 500 to 4000 cm^−1^, a strong peak observed at 759 cm^−1^ for pure NiCo and at 732 cm^−1^ for NiCo CTAB and NiCo L-lysine correlated to M-O stretching vibrations mode. The peaks at 3719 and 3614 cm^−1^ correspond to O-H stretching vibrations for NiCo, NiCo CTAB and NiCo L-lysine respectively. Only metal (Ni and Co) vibrational peak is detected in the sample; no other peaks for surfactants (CTAB, and L-lysine) are revealed, depicting the complete conversion of metal salts into metal oxides after the calcination process. The absence of any other peak suggests the high purity of the NiCo_2_O_4_ phase.^35^^,^^36^^,^^37^

**Figure 2:**
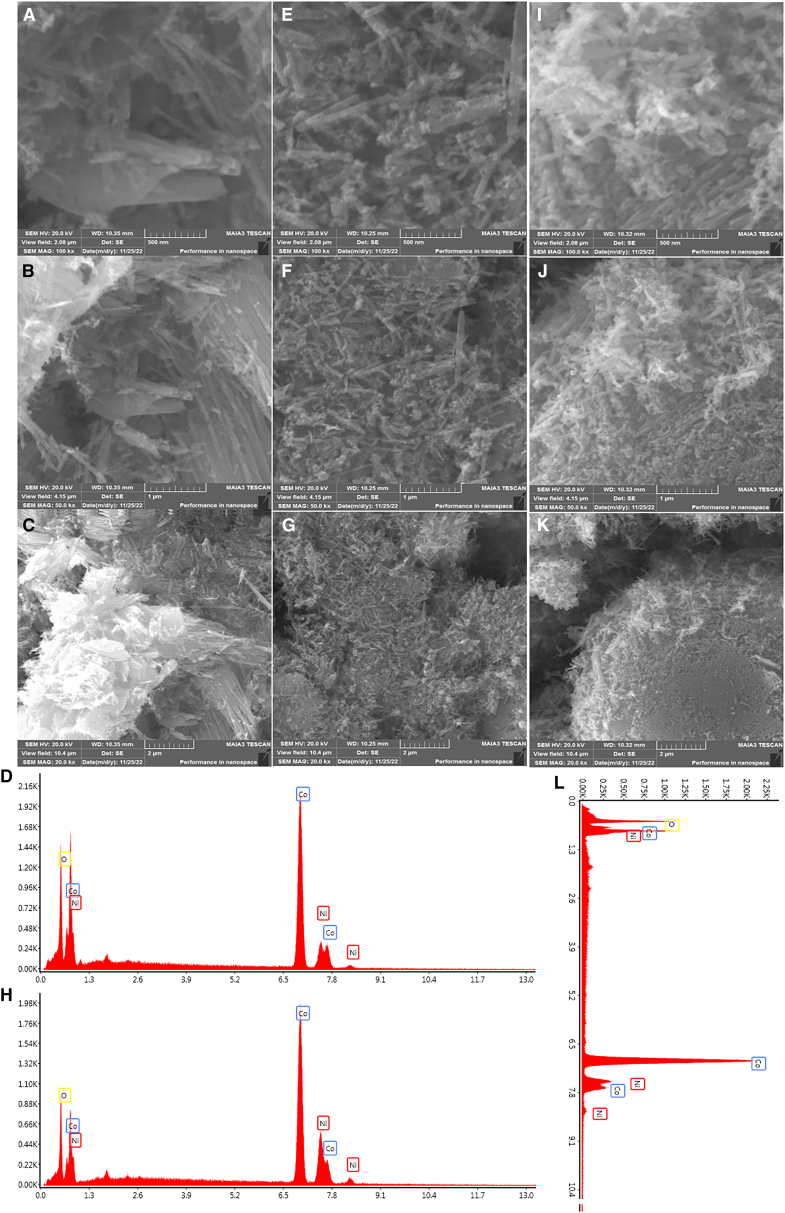
SEM images at different resolutions of 500 nm, 1 μm and 2 μm along with EDX profile (A–D) NiCo. (E–H) NiCo CTAB. (I–L) NiCo L-lysine.

The morphology of NiCo, NiCo CTAB and NiCo L-lysine was analyzed by SEM analysis at different magnifications (Figure 2). The SEM micrographs illustrated highly dense and very fine needles uniformly distributed over a nickel foam substrate. The shape of the metal oxides is highly contingent on the surfactant utilized.^38^ The pure NiCo oxides exhibit a needle shape that is stacked uniformly and has an average diameter of 37.5 nm. The NiCo oxides stabilized by CTAB disclosed small needles distributed randomly while L-lysine stabilized NiCo oxides displayed immensely condensed elongated needles at the edges. Their average diameter is calculated to be as 31.15 and 30.55 nm, respectively (Figure S1). The particles are highly pure and without agglomeration.^39^ The L-lysine assisted needles morphology supports water electrolysis reaction with more efficacity. The elemental composition of the prepared oxides was determined through EDX analysis (Figure 2). The EDX profile of all the samples of NiCo, NiCo CTAB, and NiCo L-lysine consists of nickel, cobalt and oxygen atoms only. No other peak for surfactants is observed thus further confirming their purity. The reaction mechanism of NiCo oxide fabrication is assumed to be followed by this pathway (Equations 6, 7, 8, 9, and 10).^40^

(Equation 6) $$\text{Co}{\left({\text{NH}}_{2}\right)}_{2}+H_{2}O\;⟶\;2NH_{3}+CO_{2}$$

(Equation 7) $$CO_{2}+H_{2}O\;⟶\;{\text{CO}}_{3}^{-2}+{\;H}^{+}$$

(Equation 8) $$NH_{3}+H_{2}O⟶\;{\text{NH}}_{4}^{+}+{\text{OH}}^{-}$$

(Equation 9) $$Ni^{+2}+Co^{+2}+{\text{OH}}^{-}+{\text{CO}}_{3}^{-2}+H_{2}O\;⟶\;\text{NiC}o_{2}\;{\left(\text{OH}\right)}_{3}\;{\left(CO_{3}\;\right)}_{1.5}\;H_{2}O$$

(Equation 10) $$\text{Ni}{\text{Co}}_{2}{\left(\text{OH}\right)}_{3}{\left({\text{CO}}_{3}\right)}_{1.5}H_{2}O+O_{2}\to \text{Ni}{\text{Co}}_{2}O_{4}+{\text{CO}}_{2}+H_{2}O$$

Urea is utilized as an alkali source. Ni^+2^ and Co^+2^ ions combined with hydroxyl (${\text{OH}}^{-})$ and carbonate $\left({\text{CO}}_{3}^{-2}\right)$ ions to yield needles-shaped NiCo carbonate hydroxide which on calcination changes to oxide structure.^41^ In addition, the existence of $F^{-}$ in the NH_4_F promotes the formation of nanoneedles by activating the substrate surface and hence provides more active sites for nucleation and crystal growth.^42^ Adding more, surfactants (CTAB and L-lysine) also aids in changing the crystallite size and shape of NiCo oxides as demonstrated by SEM images.^43^

### Hydrogen evolution reaction (HER)

**Figure 3:**
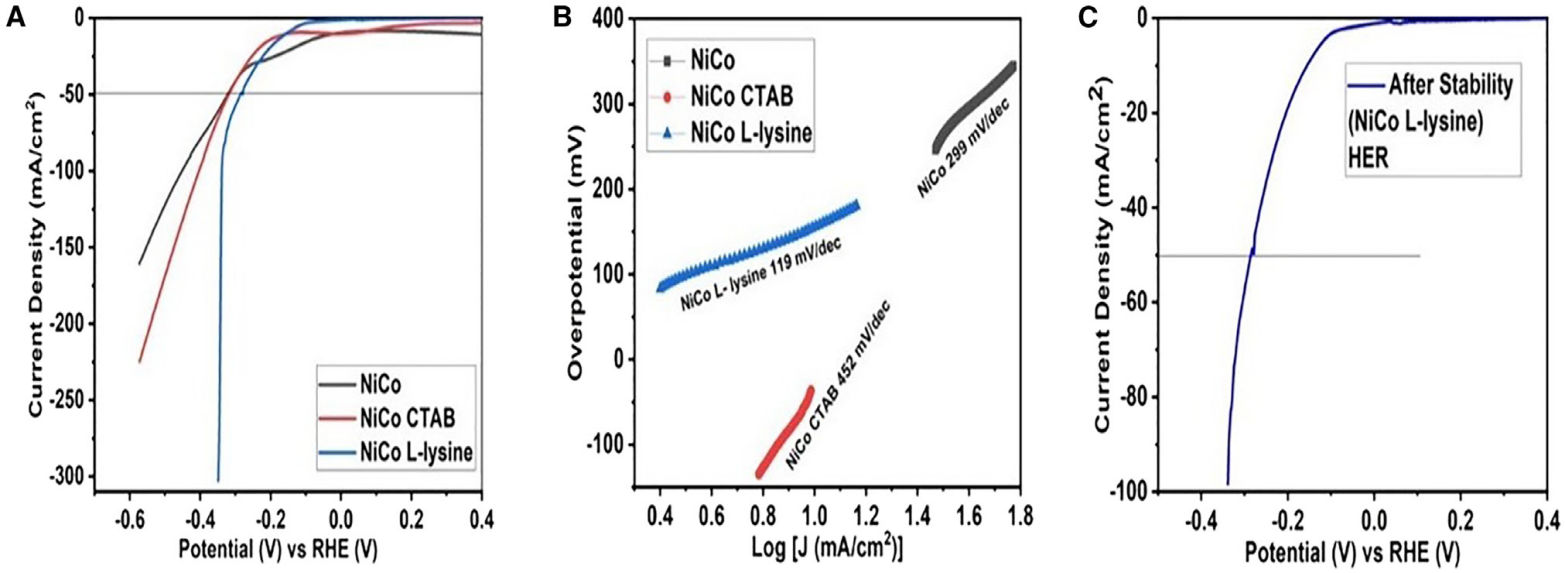
HER activity parameters (A) LSV polarization curve. (B) Tafel slope values. (C) Stability check after 500 CV cycles.

The efficacy of the prepared NiCo oxides was assessed for HER under an alkaline medium (1 M KOH electrolyte). The LSV analysis for all the catalysts was performed by applying negative potential under identical conditions (Figure 3A). The results revealed the lowest onset potential of 83 mV for NiCo L-lysine in comparison to pure NiCo (246 mV) and NiCo CTAB (134 mV). The onset potential is described as the potential needed to attain a current density of 1 mA/cm^2^. The catalysts that depicted low onset potential value tend to enhance electro-catalytic execution.^30^ The catalyst NiCo L-lysine attains a current density of 50 mA/cm^2^ at the expense of 286 mV potential thus exhibiting as the best HER activating material. However, the pure NiCo and NiCo CTAB achieved the same current density of 50 mA/cm^2^ at the expenditure of 320 and 318 mV potential, respectively. The LSV results strongly reflected that surfactants greatly assist in better current density output with the lowest potential values. Noticeably, the HER activity of NiCo L-lysine catalyst is well correlated and comparable to the reported work (Table S1).

The catalytic activity of NiCo oxides was further evaluated by computing Tafel slope values from the LSV polarization curves at the onset of HER potential. The Tafel slope parameter is crucial in dictating the kinetics and the rate-determining step of the electrolytic reaction. Generally, HER reaction follows specific pathways involving hydrogen adsorption and desorption through the Volmer, Heyrovsky and Tafel steps (Equations 11, 12, and 13).

(Equation 11) $$H_{2}O+e^{-}\;\to H_{ads}+{OH}^{-}\;\left(\text{Volmer}\;\;\text{step}\right)$$

(Equation 12) $$H_{ads}+H_{2}O+e^{-}\to \;H_{2}+{OH}^{-}\;\left(\text{Heyrovsky}\;\;\text{step}\right)$$

(Equation 13) $$H_{ads}+H_{ads}\to H_{2}\;\left(\text{Tafel}\;\;\text{step}\right)$$

The rate-determining, is highly dependent on the values of the Tafel slope. Therefore, the Tafel slope values of 120, 40 and 30 mV/dec represented Volmer, Heyrovsky, or Tafel steps, respectively.^44^^,^^45^ In this work, the lowest Tafel slope value of 119 mV/dec for NiCo L-lysine in comparison to other catalysts, evinced faster kinetics and the reaction followed the Volmer step for the hydrogen generation (Figure 3B). The mass activity which is stated as the current density per unit mass loading of the catalyst (mass activity = j (mAcm^−2^)/m (mgcm^−2^)) at certain potential (1.5V–300 mV) is assessed as 23.26, 17.04 and 16.62 Ag^-1^ for NiCo L-lysine, NiCo CTAB and NiCo, respectively. The term “mass activity” implies that all atoms within particles act as electrocatalytic active sites. Catalysts with smaller sizes tend to exhibit higher mass activity due to their larger surface area and higher number of surface atoms (as depicted by NiCo L-lysine).^46^

Finally, the durability test for NiCo L-lysine was conducted by running 500 CV cycles (0 to 1V vs. RHE V) and results expressed better stability and retained initial activity (Figure 3C). The outstanding HER activity is attributed to the morphological variations imposed by applying different surfactants which helps in creating smaller size particles with more active sites and surface area. In addition, the direct growth of the catalysts on nickel foam substrate also enables fast charge transfer along with metals harmonious effect and hence leading to higher HER. Thus, these modifications are helpful in the development of good electro-catalysts with outstanding output.^47^

### Oxygen evolution reaction (OER)

**Figure 4:**
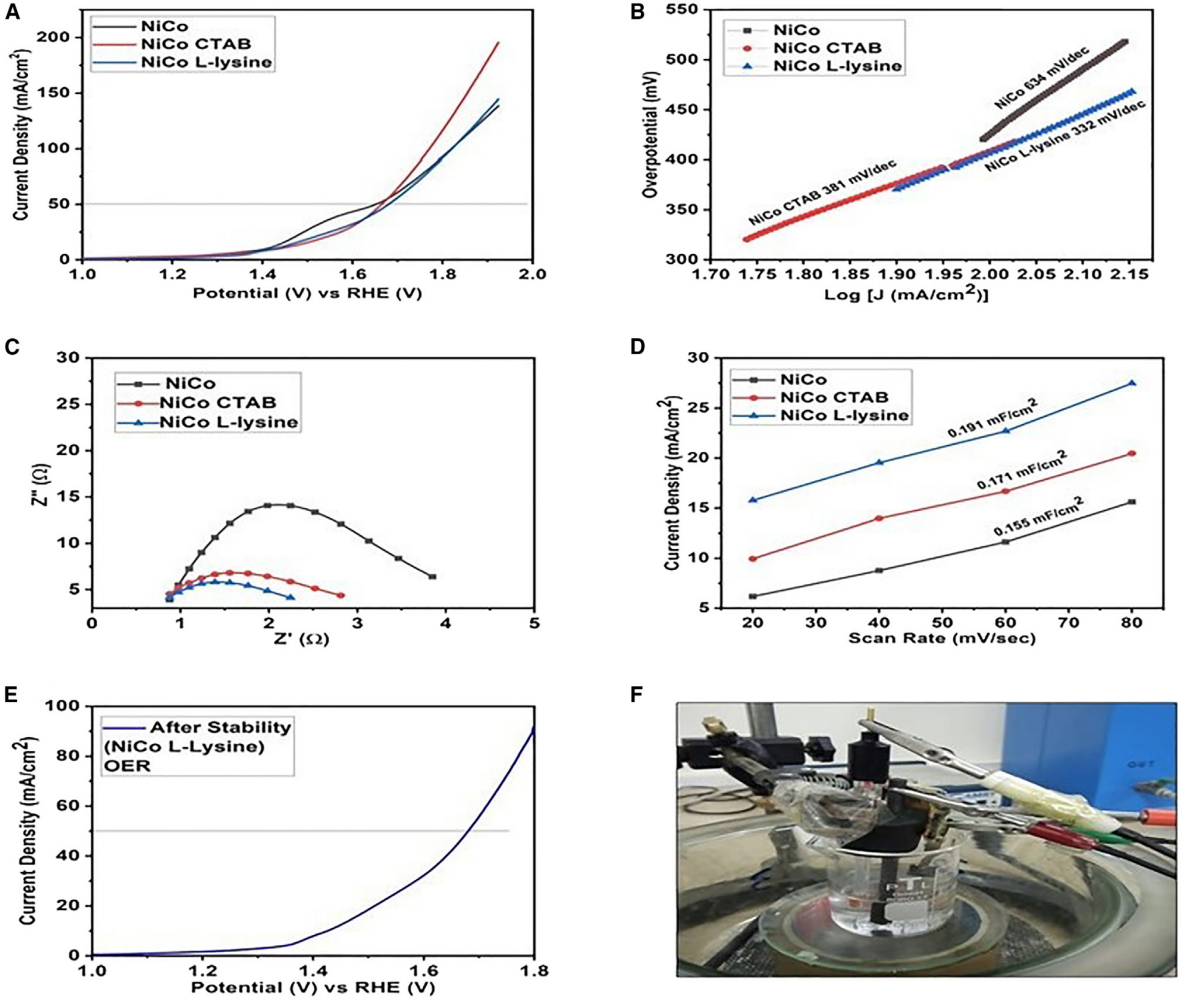
OER activity parameters (A) LSV polarization curve. (B) Tafel slope values. (C) Nyquist plot. (D) Double layer capacitance values. (E) Stability check after 500 CV cycles. (F) Potentiostat setup.

The electro-catalytic OER performance for the prepared NiCo oxides was evaluated using 1 M KOH and a standard three-electrode setup (Figure 4F). The linear sweep voltammetry curves (LSV) exhibited the lowest onset potential for NiCo L-lysine at 130 mV, while NiCo and NiCo CTAB displayed the OER process initiated at 420 and 150 mV respectively (Figure 4A). Nonetheless, the catalysts NiCo, NiCo CTAB and NiCo L-lysine needed an overpotential of 420, 430, and 450 mV respectively to acquire a current density of 50 mA/cm^2^ for OER. Usually, the efficacy of any electro-catalyst is displayed by an overpotential value calculated at the current density of 10 mA/cm^2^. Here in this work, nickel foam has been employed as an electrode substrate. The surface of nickel foam got oxidized (Ni^+3^ to Ni^+4^) at 10 mA/cm^2^, so, to avoid any interruptions by this oxidation peak, the overpotential values are normally computed at 50 mA/cm^2^.^48^ This excelling behavior of NiCo L-lysine and NiCo CTAB could be justified by proper surfactant incorporation and smaller particle size.^49^ The details of comparative electro-catalytic activity for different materials are mentioned in the table (Table S1). These results further resembled with Tafel slope values of all the catalysts and justify their kinetics based on their nanoscale size and synergistic metals interplay. The Tafel slope value for NiCo L-lysine was enumerated to be 332 mV/dec, while NiCo and NiCo CTAB manifested Tafel slopes of 634 and 381 mV/dec respectively, undeniably specifying better performance for NiCo L-lysine catalyst (Figure 4B). The OER execution can also be credited to the co-existence of Ni^+3^/Ni^+2^ and Co^+3^/Co^+2^ in NiCo_2_O_4_ as reported in the literature. Herein, the Co element was found to be more active in collation with Ni and thus interacted with water molecules to form NiCo-O bonding to bring cleavage of water.^12^^,^^50^ The proposed OER pathway under an alkaline medium is presented in Equations 14, 15, 16, 17, and 18.

(Equation 14) $$M^{*}+{OH}^{-}\;\to {M-OH}^{*}+e^{-}$$

(Equation 15) $${M-OH}^{*}+{OH}^{-}\;\to {M-O}^{*}+e^{-}$$

(Equation 16) $${2M-O}^{*\;}\to 2M^{*\;}+O_{2}$$

(Equation 17) $${M-O}^{*}+{OH}^{-}\to \;{M-OOH}^{*}+e^{-}$$

(Equation 18) $${M-OOH}^{*}+{OH}^{-}\;\to \;M^{*}+e^{-}+O_{2}+H_{2}O$$

In these equations, M^∗^ represented the metal active site while O^∗^, OH^∗^ and OOH ^∗^ symbolized adsorbed intermediates for OER. There are two possible ways to produce oxygen. The simplistic approach involves the direct combination of two M-O^∗^ intermediates to generate O_2_ (Equation 22. Conversely, in another method, M-O^∗^ links with OH^−^ to form M-OOH^∗^, which eventually yields O_2_ (Equations 23 and 24). Moreover, the oxides based on transition metals follow a pathway involving the formation of hydroxide intermediates (Equation 18).^45^

Moreover, the kinetics was also investigated from the charge transfer resistance (R_ct_) utilizing electrochemical impedance spectroscopy (EIS) at 0.65V (Figure 4C). The semicircle of the Nyquist plots at low frequency region gives the charge transfer resistance values as 3.055, 1.936 and 1.366 Ω for NiCo, NiCo CTAB and NiCo L-lysine, respectively. Notably, the lower charge transfer resistance for NiCo L-lysine stipulated its faster kinetics and better OER-performing activity. The exchange current density is also a vital parameter to ascertain charge transfer at the electrode and electrolyte interface. The NiCo L-lysine has a large exchange current density of 4.69 mA/cm^2^ in comparison to NiCo (2.10 mA/cm^2^) and NiCo CTAB (3.31 mA/cm^2^), respectively. This large value of exchange current density also confirmed the higher charge transfer at the interface for the OER process.^51^ The mass activity value (at 1.5 V) for NiCo L-lysine was found to be 7.97 Ag^-1^, higher than NiCo CTAB (6.67 Ag^-1^) and simple NiCo (5.960 Ag^-1^) clearly depicting its better catalytic activity.^52^

The double layer capacitance (C_dl_) of the catalysts was measured by taking CV cycles in the non-Faradaic region (−0.1 to 0.2V) at various scan rates of 20, 40, 60, and 80 mV/s (Figure S2). The increase in current density with increasing scan rates indicated that the catalysts have capacitive behavior.^53^ The NiCo L-lysine has the highest double-layer capacitance value of 0.191 mF/cm^2^. The NiCo and NiCo CTAB demonstrated 0.155 and 0.171 mF/cm^2^ respectively (Figure 4D). The corresponding electrochemical surface area (ECSA) for all the catalysts was also calculated and found to be higher for NiCo L-lysine (4.775 cm^2^) comparatively as NiCo (3.875 cm^2^) and NiCo CTAB (4.275 cm^2^).

The relative ECSA of NiCo L-lysine was estimated by using the following relation (Equation 19).

(Equation 19) $$Relative\;ECSA=\frac{ECSA\;\left(NiCO\;L-lysine\right)}{ECSA\;\left(NiCo\right)}$$

The relative ECSA of NiCo L-lysine was concluded to be 1.23 times higher than the pure NiCo oxides indicating comparatively abundant accessible active sites directing to enhanced activity of the catalyst.^30^

The long-term durability of the catalysts is considered an obstacle to its commercialization. Therefore, a long-standing electrolysis test was conducted to determine its strength. The stability test implemented for 500 CV cycles exhibits no noticeable decrease in the current density with increasing potential thus ensuring the recycling capability of the NiCo L-lysine (Figures 4E and S3). These results undoubtedly suggested that surfactants have succeeded much in increasing OER performance and this kind of synthesis is highly recommendable for future energy production applications.

### Conclusion

The synthesis of NiCo bimetallic oxides was achieved via the hydrothermal method in the presence of CTAB and L-lysine. The catalytic performance of the resulting bifunctional catalyst for both HER and OER under basic conditions (pH 14, 1 M KOH) was assessed. The synthesized NiCo oxides underwent comprehensive characterization through X-ray diffraction, Fourier transform infrared spectroscopy, and scanning electron microscopy coupled with energy-dispersive X-ray techniques. The CTAB and L-lysine affected the morphology of NiCo oxides significantly. The water-splitting reaction was studied concerning morphological variations induced and it was found that NiCo L-lysine surpassed NiCo CTAB in catalyzing the process. The NiCo L-lysine needed an overpotential of 286 and 450 mV to attain a current density of 50 mA/cm^2^ and the corresponding Tafel slope values were computed as 119 and 332 mV/dec for HER and OER, respectively. These results suggest that the NiCo prepared in the presence of L-lysine has the potential to catalyze the electrochemical water splitting reaction, which could be utilized for energy production purposes.

## Resource availability

### Lead contact

Further information and requests for resources should be directed to and will be fulfilled by the lead contact, Munawar Iqbal (bosalvee@yahoo.com).

### Materials availability

This study did not generate new unique reagents.

### Data and code availability

• This study did not generate any datasets. The lead contact will share all data reported in this paper upon request.
• This paper does not report the original code.
• Any additional information required to reanalyze the data reported in this paper is available from the lead contact upon request.

## Acknowledgments

The authors are thankful to the Deanship of Graduate Studies and Scientific Research at University of Bisha for supporting this work through the Fast-Track Research Support Program. The authors express their gratitude to Princess Nourah bint Abdulrahman University Researchers Supporting Project number (PNURSP2024R11), Princess Nourah bint Abdulrahman University, Riyadh, Saudi Arabia. The authors would like to thank the anonymous reviewers for their valuable suggestions for improving the manuscript.

## Author contributions

S.A. conceived the study. Abid Ali (author 4) and A. T. designed the experiments and wrote original draft. Abid Ali (author 5) performed the experiments. A.N. and W.M. review and edited the manuscript with input from all coauthors. N.A. administrated the project and helped in formal analysis by providing resources. M.I. visualized, validated the data and helped in review and editing.

## Declaration of interests

The authors declare no competing interests.

## STAR★Methods

### Key resources table

**Table:** 

REAGENT or RESOURCE	SOURCE	IDENTIFIER
**Chemicals, peptides, and recombinant proteins**		

Nickel nitrate hexahydrate	Sigma-Aldrich	72253
Cobalt nitrate hexahydrate	Sigma-Aldrich	230375
Urea	Sigma-Aldrich	U5128
Cetyl Trimethyl Ammonium Bromide	Sigma-Aldrich	219374
L-lysine	Sigma-Aldrich	P6516
hydrochloric acid	Sigma-Aldrich	258148
Potassium hydroxide	Sigma-Aldrich	221473
Ethanol	Sigma-Aldrich	493511
Acetone	Sigma-Aldrich	179124
Nickel foam	Sigma-Aldrich	ESYNTH021

**Software and algorithms**		

Origin	Origin 2021	https://www.originlab.com/
EChem Analyst	EChem 7.8.4	https://www.gamry.com/

**Others**		

Potentiostat	GAMRY/1000E	https://www.gamry.com/
Hg/HgO Electrode	SKU 932–00008	https://www.gamry.com/
Graphite electrode	SKU 935–00003	https://www.gamry.com/

### Method details

#### Synthesis of NiCo oxides

Typical hydrothermal synthesis to fabricate NiCo oxides directly on Ni foam involved the following steps (Figure 1A). The nickel foam was cut into pieces of 1.5 cm × 1 cm dimension and washed with 3M HCl, acetone, ethanol and deionized water ultrasonically for 10 min each. It was then oven-dried at 100°C and kept for future use. Ni and Co with an equal molar ratio of 5 mmol were mixed with 12 mmol urea and 6 mmol ammonium fluoride in 50 ml deionized water as a solvent and kept under magnetic stirring for 30 min. A fixed amount (0.05 g) of surfactants CTAB and L-lysine was also added. Then the reddish solution was poured into a 100 ml Teflon-lined stainless steel autoclave with nickel foams inside and transferred into the oven which was then heated at 150°C for 5 hours. After completion of the reaction, the autoclave was taken out from the oven and cooled down at room temperature. Then foams were washed with ethanol and water and dried in an oven at 80°C for 3 hours. Finally, the product was calcined in a muffle furnace at 300°C for 2 hours. For comparison, the pure NiCo oxides were also prepared by the same route instead addition of surfactants. The samples were denoted as pure NiCo, NiCo CTAB, and NiCo L-lysine (by the name of surfactants). The loading amounts of metal oxides have been calculated as approximately ∼2.3 mg/cm^2^. For this, nickel foam was weighed before and after the hydrothermal reaction.

#### Characterization

All the synthesized NiCo oxides were characterized by a Scanning electron microscope (MAIA 3 Tescan) equipped with an EDX detector, Fourier transform infrared spectrometer (Nexus 670 FTIR Nicolet USA), and powder X-Ray diffractometer (D2 Phaser- Bucker).

#### Electrochemical study

The electrochemical measurements were performed in 1 M KOH solution using potentiostat interface-1000E of GAMRY instruments, USA at room temperature. The prepared NiCo oxides were employed as working electrodes along with Graphite rod and Hg/HgO as counter and reference electrodes. The electrochemical active surface area of working electrodes was 1cm^2^. All the recorded experimental potentials were converted to reversible hydrogen electrode scale (RHE) without iR correction using standard Nernst Equation 20.

(Equation 20) $$E_{\text{RHE}}=E_{\text{Hg}/\text{HgO}}+\left(0.0591*\text{pH}\right)+0.098$$

The pH value for 1M KOH was 14. The HER and OER activities of the NiCo oxides were analyzed by the linear sweep voltammetry (LSV) polarization curves at a scan rate of 5mVsec^-1^ (0 to ±1.5V). The overpotential values were estimated by the Equations 21 and 22.

(Equation 21) $$\eta_{OER}=E_{\text{RHE}}-1.23\;V$$

(Equation 22) $$\eta_{HER}=E_{RHE}-0\;V$$

The current density value was standardized according to the electrode’s surface area. The Tafel slope was determined from LSV polarization curves by plotting overpotential (η) values to the log of current density (*j*) by applying Equation 23.

(Equation 23) η=blog(j)+a

Where b is the Tafel slope and a is a constant value. The electrochemical impedance spectroscopy (EIS) was carried out in the frequency range of 10 mHz to 100 kHz at 0.65V to find the charge transfer resistance (R_ct_). Double layer capacitance (C_dl_) was computed from the non-Faradaic region (-0.1 to 0.2V) in the polarization curve at different CV cycles having scan rates of 20 to 80 mV/sec. From double-layer capacitance, the electrochemical surface area (ECSA) was assessed by using Equation 24.

(Equation 24) $$\text{ECSA}=\frac{C_{\text{dl}}}{C_{s}}$$

Where Cs, the specific capacitance, has the value of 0.04 mFcm^-2^, as mentioned in the literature. The exchange current density value was figured out by using Equation 25.

(Equation 25) $$I_{\text{ex}}=\text{RT}/\text{nF}Ɵ$$

Where R is the universal gas constant (8.314 J/mol.K), T is the reaction temperature (298 K), n is the number of electrons transferred (2e^-^ for HER, 4e^-^ for OER), Ɵ is the charge transfer resistance (R_ct_), and F is the Faraday constant (96,485 C/mol).^51^^,^^54^^,^^55^^,^^56^

(R_ct_), and F is the Faraday constant (96,485 C/mol).^51^^,^^54^^,^^55^^,^^56^
