# Supplementary material for: L-lysine and surfactant-assisted synthesis of NiCo bimetal oxides for electrochemical water splitting
Source: iScience. 2024 Aug 31;27(12):110823. doi: 10.1016/j.isci.2024.110823 (PMC11626774; doi:10.1016/j.isci.2024.110823)
Supplement: Document S1. Figures S1–S3 and Table S1 [file mmc1.pdf]

iScience, Volume *27*

Supplemental information

L-lysine and surfactant-assisted synthesis of NiCo bimetal oxides for electrochemical water splitting

Anila Tabassum, Sadia Ata, Norah Alwadai, Wissem Mnif, Abid Ali, Abid Ali, Arif Nazir, and Munawar Iqbal

iScience

Supplemental information

L-lysine and surfactant-assisted synthesis of NiCo bimetal oxides for electrochemical water splitting

Anila Tabassum, Sadia Ata, Norah Alwadai, Wissem Mnif, Abid Ali, Abid Ali, Arif Nazir, Munawar Iqbal

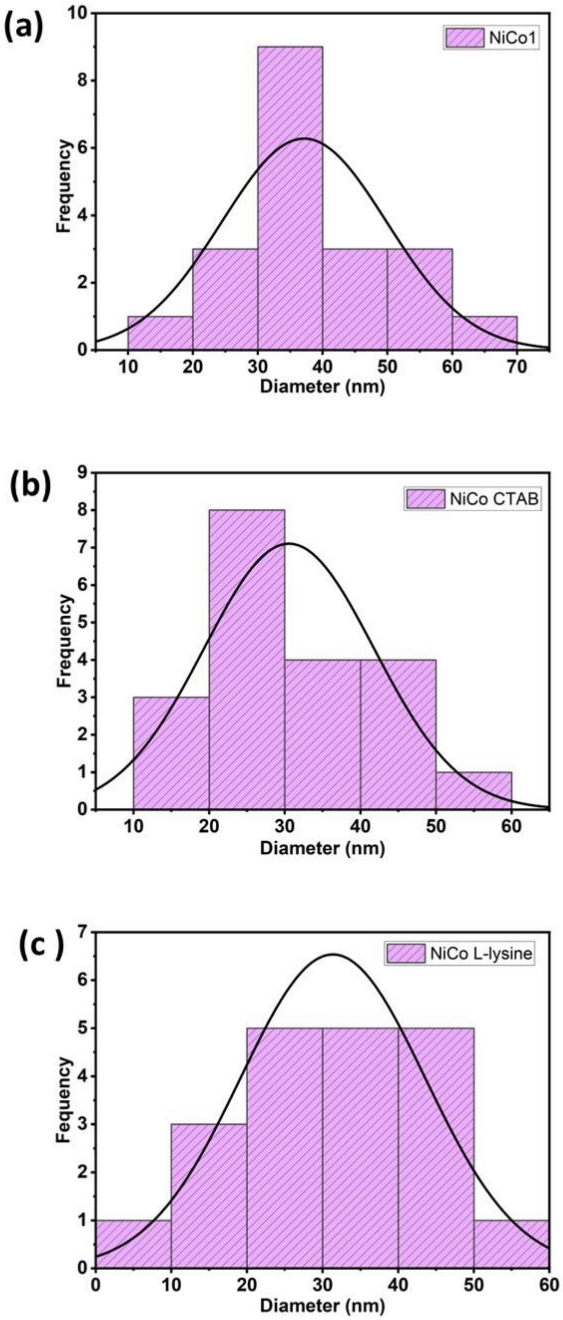

Figure S1: Histogram showing the particle size distribution, Related to Figure 2.

Histogram for the particle size distribution of (a) NiCo oxides, (b) with NiCo-CTAB and (c) NiCo-L-lysine.

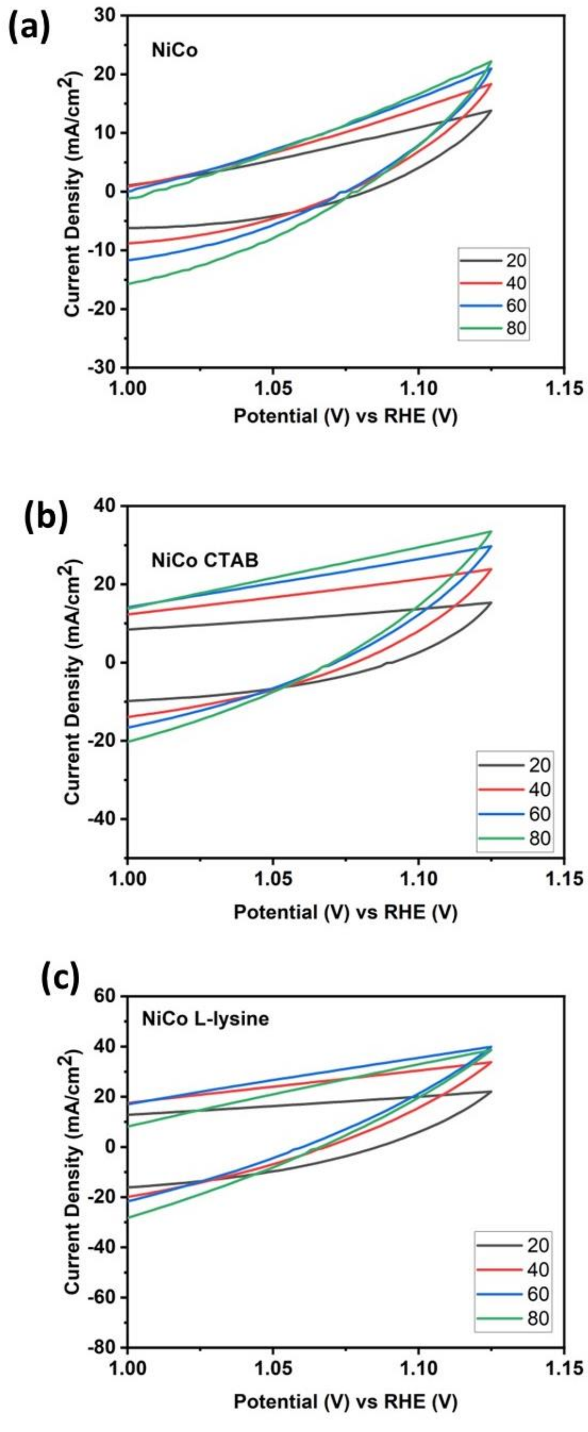

Figure S2: CV curves of the synthesized bimetallic materials for the assessment of double layer capacitance, Related to Figure 4(d).

CV curves for the (a) NiCo oxides, (b) with NiCo-CTAB and (c) NiCo-L-lysine at different scan rates (20-80 mVs^{-1}).

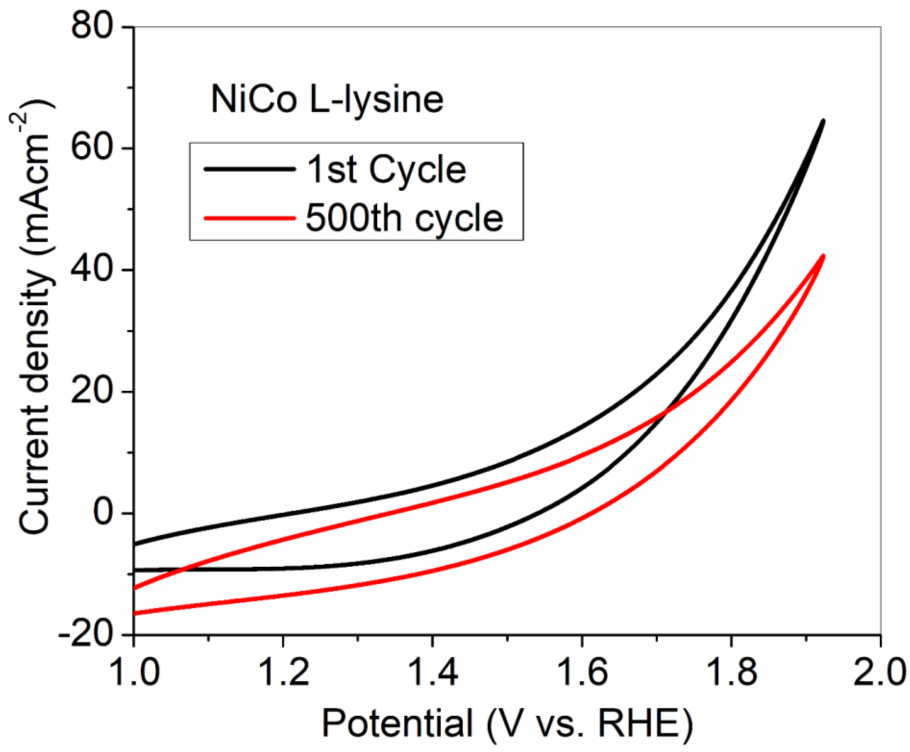

Figure S3: Cyclic stability of electrocatalyst, Related to Figure 3(c).

CV curve for the NiCo-L-lysine based electrode at the 500 cycles

Table S1: Different electrochemical parameters for the comparison of NiCo oxide catalysts with literature**, Related to Figure 4.**

Catalyst	HER Overpotential	OER Overpotential	Ref.
**$NiCo_2O_4$**		379 mV @10 mA/cm^2	[1]
**Amorphous cobalt-nickel-phosphide**	92 mV@10 mA/cm^2	277 mV @10 mA/cm^2	[2]
**Nickel-cobalt oxide/sulfide/phosphide composite nanowire**	143 mV @10 mA/cm^2	254 @10 mA/cm^2	[3]
**Ni/Co metal-organic framework (MOF)**	139 mV@10 mA/cm^2	290 @10 mA/cm^2	[4]
**SnS/NiCo2O4**	154 mV @20 mA/cm^2	302 mV@20 mA/cm^2	[5]
**Co–Ni bimetallic phosphides**	117@10 mA/cm^2	272@10 mA/cm^2	[6]
**NiPcCOF**		460 @50 mA/cm^2	[7]
**Nickel (Ni) doped $CuCoO_2$**		409 mV@10 mA/cm^2	[8]
**$NiFe_2O_4$ powders**		410 mV @10 mA/cm^2	[9]
**CoO/NiO/NF**		467 mV @50 mA/cm^2	[10]
**NiCo L-lysine**	83 mV onset and 320 @50 mA/cm^2	130mV onset and 430@50 mA/cm^2	This work

References

1. Ndambakuwa, W., Ndambakuwa, Y., Choi, J., Fernando, G., Neupane, D., Mishra, S.R., Perez, F., and Gupta, R.K. (2021). Nanostructured nickel-cobalt oxide and sulfide for applications in supercapacitors and green energy production using waste water. Surface and Coatings Technology *410*, 126933.
2. Chai, L., Liu, S., Pei, S., and Wang, C. (2021). Electrodeposited amorphous cobalt-nickel-phosphide-derived films as catalysts for electrochemical overall water splitting. Chemical Engineering Journal *420*, 129686.
3. Yan, F., Yan, L., Wei, X., Han, Y., Huang, H., Xu, S., Liang, X., Zhou, W., and Guo, J. (2022). Structure-design and synthesis of nickel-cobalt oxide/sulfide/phosphide composite nanowire arrays for efficient overall water splitting. International Journal of Hydrogen Energy *47*, 10616-10627.
4. Van Phuc, T., Jana, J., Ravi, N., Kang, S.G., Chung, J.S., Choi, W.M., and Hur, S.H. (2022). Highly active Ni/Co-metal organic framework bifunctional electrocatalyst for water splitting reaction. International Journal of Hydrogen Energy *47*, 22787-22795.
5. John, G., Gopalakrishnan, S., Sharan, A., Navaneethan, M., Kulandaivel, J., Singh, N., and Justin Jesuraj, P. (2022). Exploring the Heterostructure Engineering of SnS/NiCo2O4 for Overall Water Splitting. Energy & Fuels.
6. Chu, H., Feng, P., Jin, B., Ye, G., Cui, S., Zheng, M., Zhang, G.-X., and Yang, M. (2022). In-situ release of phosphorus combined with rapid surface reconstruction for Co–Ni bimetallic phosphides boosting efficient overall water splitting. Chemical Engineering Journal *433*, 133523.
7. Jarju, J., Díez, A., Frey, L., Sousa, V., Carbó-Argibay, E., Gonçalves, L., Medina, D., Lebedev, O., Kolen'Ko, Y.V., and Salonen, L. (2022). Synthetic strategy for metallophthalocyanine covalent organic frameworks for electrochemical water oxidation. Materials Today Chemistry *26*, 101032.
8. Yang, M., Han, N., Shi, L., Gao, H., Liu, X., Mi, Y., Zeng, X., Bai, J., and Xiong, D. (2022). Effect of nickel doping on the structure, morphology and oxygen evolution reaction performance of Cu-BTC derived CuCoO 2. Dalton Transactions *51*, 8757-8765.
9. Simon, C., Timm, J., Tetzlaff, D., Jungmann, J., Apfel, U.P., and Marschall, R. (2021). Mesoporous NiFe2O4 with tunable pore morphology for electrocatalytic water oxidation. ChemElectroChem *8*, 227-239.
10. Oh, Y., Theerthagiri, J., Kumari, M.A., Min, A., Moon, C.J., and Choi, M.Y. (2024). Electrokinetic-mechanism of water and furfural oxidation on pulsed laser-interlaced Cu2O and CoO on nickel foam. Journal of Energy Chemistry *91*, 145-154.
